# High prevalence of MDR and XDR *Escherichia coli* in hospital wastewater from Shiraz, Iran: ESBL and carbapenemase production

**DOI:** 10.1186/s12866-025-04398-2

**Published:** 2025-10-09

**Authors:** Abolfazl Rafati Zomorodi, Mohammad Reza Samaei, Himen Salimizand, Mohammad Hassan Parvizi Mashhadi, Mohammad Motamedifar

**Affiliations:** 1 Department of Bacteriology and Virology, School of Medicine, Shiraz University of Medical Sciences, Shiraz, Iran; 2 Education Development Center, Student Committee of Medical Education Development, Shiraz University of Medical Sciences, Shiraz, Iran; 3 Department of Environmental Health Engineering, School of Public Health, Shiraz University of Medical Sciences, Shiraz, Iran; 4 Vaccine and Infectious Disease Organization Saskatoon, Saskatoon, SK Canada; 5 Department of Vaccinology and Immunotherapeutics, School of Public Health, University of Saskatchewan, Saskatoon, SK Canada; 6 HIV/AIDS Research Center, Institute of Health, Shiraz University of Medical Sciences, Shiraz, Iran

**Keywords:** E. coli, ESBL, MDR, XDR, Phylogroups, Sequence types

## Abstract

**Background:**

Hospital wastewater (HWW) is a significant reservoir for multidrug-resistant (MDR) bacteria and antimicrobial resistance (AMR) genes, posing serious public health risks. This study investigated the phenotypic and genotypic resistance profiles of *Escherichia coli* (*E. coli*) isolates from HWW in Shiraz, Iran. Thirty-six HWW samples (18 influent, 18 effluent) were collected weekly over six weeks from three major hospitals. *E. coli* isolates were identified and assessed for antimicrobial susceptibility, extended-spectrum β-lactamase (ESBL) and carbapenemase production, resistance genes, phylogroups, and sequence types (STs).

**Results:**

A total of 68 *E. coli* isolates were obtained (33 influent, 35 effluent). High resistance rates were observed for ampicillin (97.1%), cefazolin, and amoxicillin/clavulanic acid (86.8%). The lowest resistance rates were to imipenem (5.9%), meropenem (13.2%), and chloramphenicol (19.1%). MDR and extensively drug-resistant (XDR) profiles were identified in 80.9% and 22.1% of isolates, respectively. ESBL production was found in 57.4% of isolates. All nine carbapenem-resistant isolates were tested by both modified carbapenem inactivation method (mCIM) and EDTA-CIM (eCIM); eight (88.9%) were positive by mCIM, while all were negative by eCIM. Resistance genes detected included *bla*TEM (39.7%), *bla*CTX-M (33.8%), and *bla*SHV (2.9%). Three isolates carried *bla*OXA-48, while one carried *bla*IMP and another carried *bla*VIM. Phylogroup B2 was most frequent (22.1%), with ST131 being the dominant type (33.8%).

**Conclusion:**

In conclusion, HWW may serve as a potential reservoir for resistant and pathogenic *E. coli* isolates, indicating the importance of monitoring wastewater as a step in addressing the issue of AMR.

**Supplementary Information:**

The online version contains supplementary material available at 10.1186/s12866-025-04398-2.

## Background

The World Health Organization (WHO) has identified antimicrobial resistance (AMR) as one of the ten most severe global health threats [1]. Despite ongoing efforts to combat AMR, the estimated global death toll from infections caused by multi-drug resistant (MDR) bacteria reached 4.9 million in 2019, particularly affecting low- and middle-income countries [2]. AMR is notably driven by selection pressure resulting from the widespread use of antibiotics, which has created significant quantities of antibiotic residues in various environments. For instance, multiple studies have shown that antibiotics discarded into wastewater do not degrade effectively [3–5]. Consequently, wastewater, particularly hospital wastewater (HWW), has emerged as critical reservoirs for the dissemination of MDR bacteria and antimicrobial resistance genes (ARGs).

*Escherichia coli* (*E. coli*) is a member of the ESKAPE group, which includes *Enterococcus* spp., *Staphylococcus aureus*, *Klebsiella pneumoniae*, *Acinetobacter baumannii*, *Pseudomonas aeruginosa *[6]. These bacteria have been identified by the European Centre for Disease Prevention and Control (ECDC) as significant contributors to rising mortality and morbidity rates, primarily attributed to their high levels of AMR [7]. Recent systematic reviews have indicated that *E. coli *is responsible for over 500,000 deaths worldwide each year [2, 8].

One of the most significant mechanisms of resistance observed in Gram-negative bacteria is the production of extended-spectrum β-lactamases (ESBLs) [9, 10]. Notably, the prevalence of ESBL-producing *E. coli* (ESBL-EC) strains has surged, with more than 50% of *E. coli *isolates in Africa and Southeast Asian countries testing positive for ESBL production [11]. These ESBL-EC strains are predominantly MDR or even extensively drug-resistant (XDR), exhibiting resistance to many classes of antibiotics. Currently, carbapenems and colistin serve as the last line of treatment for infections caused by MDR or XDR bacteria [12]. However, the emergence of carbapenem- and colistin-resistant *E. coli *has escalated due to the widespread use of these antibiotics in both human medicine and veterinary practices [13].

*E. coli *strains exhibit significant diversity, encompassing both commensal and opportunistic pathogenic varieties. Differentiating between these strains is essential for epidemiological studies [14]. In fact, characterizing *E. coli* isolates from various sources can inform local health policies. Clermont et al. classified *E. coli *isolates into seven distinct phylogroups: A, B1, B2, C, D, and E; each phylogroup possesses specific characteristics [15]. For example, the majority of intestinal pathogenic strains are found within the B2 and D phylogroups, whereas strains more commonly associated with aquatic environments belong to phylogroups A and B1 [16].

The current study aimed to investigate the prevalence of ESBL-EC, MDR, XDR, carbapenem-resistant, and colistin-resistant *E. coli* isolates from HWW in Shiraz, southwest Iran. Additionally, it evaluated the correlation of these isolates with four main phylogroups: A, B1, B2, and D.

## Methods

### Sampling

This cross-sectional study was conducted in Shiraz, the largest medical referral city in southwestern Iran. A total of 36 HWW samples were collected from three main hospitals in Shiraz: Nemazee, Shahid Faghihi, and Hafez. Nemazee Teaching Hospital is a tertiary care facility with 914 active beds and approximately 4,500 staff. Shahid Faghihi Hospital has 485 active beds and around 2.207 staff members, while Hafez Hospital specializing in gynecology and obstetrics, has 165 active beds.

All three hospitals discharge their wastewater into on-site septic tanks, which serve as the primary treatment system by allowing sedimentation and partial anaerobic digestion of solids. No advanced biological or chemical treatment is applied before discharge. The estimated daily wastewater discharge per hospital ranges between 200 and 500 cubic meters, depending on hospital size and patient volume.

Samples were collected from both the influent and effluent of the HWW streams of each hospital using 500 mL sterile bottles, with the sample volume standardized across all collections. Grab sampling was employed, and all procedures were performed in accordance with guidelines from the WHO (2003) and national protocols issued by the Iranian Department of Environment for environmental wastewater monitoring [17]. Sampling was conducted consistently on a weekly basis over a six-week period from February 20 to March 24, 2023. Each week, one set of influent and effluent samples was collected from each hospital, resulting in a total of 36 samples (2 samples 🞨 3 hospitals 🞨 6 weeks). To minimize variation due to time-of-day or operational fluctuations, all samples were collected in the morning hours (between 8:00 and 10:00 AM). Although the study duration did not encompass multiple seasons, the sampling period covered late winter to early spring, which may help capture transitional variability in wastewater composition. Nevertheless, we acknowledge the limitation in temporal scope and recognize that seasonal patterns may influence microbial loads or resistance profiles. All samples were immediately transported in iceboxes to the Microbiology Laboratory of the School of Medicine at Shiraz University of Medical Sciences for processing.

### Samples processing

As previously described by Teban-Man et al., samples were processed through centrifugation and serial dilution for culture. Briefly, 50 mL and 100 mL of influent and effluent samples, respectively, were centrifuged (Eppendorf 5810 R, Germany) at 5,000 rpm for 20 min at 4°C. Following centrifugation, the wastewater was removed, and the sediments were resuspended using 10 mL of sterile normal saline. To prepare the serial dilution, 1 mL of the prepared suspension was transferred into new tubes containing 9 mL of sterile normal saline, resulting in dilutions from 10^−1^ to 10^−3^. Culturing was performed using the surface plate method. From the last dilution (10^−3^), 100 µL was transferred to MacConkey agar (Merck, Germany), a selective and differential medium, supplemented with 4 µg/mL ceftazidime (Sigma-Aldrich Co., St. Louis, MO, USA), in order to reduce the overgrowth of background microbiota and facilitate the isolation of *E. coli*. This concentration was not used for resistance determination but served as a selective aid. Plates were incubated at 37 °C for 24 h [18].

### Bacterial isolation and identification

From each plate, 3 to 5 pink colonies were randomly selected for further tests to identify potential *E. coli* isolates. Each colony was subjected to streak culturing on basic nutrient agar and the selective-differential Eosin Methylene Blue (EMB) medium to obtain pure single colonies, including any that exhibited a green metallic sheen. After overnight incubation, the obtained pure colonies were subjected to standard biochemical tests, including Gram staining, oxidase/catalase tests, growth patterns on Triple Sugar Iron agar (TSI), and IMViC tests (Indole, MR-VP, and Simmon’s citrate) (Merck, Germany). Presumptive *E. coli* isolates were stored in Tryptic Soy Broth (TSB) medium (Merck, Germany) supplemented with 30% glycerol at −70°C for further experiments.

### DNA extraction

Initially identified *E. coli *isolates were subjected to DNA extraction using the boiling method. As previously explained by Zomorodi et al. [12], five pure colonies from a fresh bacterial culture were picked and suspended in sterile 1.5 mL Eppendorf microtubes containing 300 µL of ultrapure distilled water. The microtubes were placed at 95 °C for 10 min and then immediately transferred to a−20°C refrigerator for 10 min. Finally, the microtubes were centrifuged at 14,000 rpm for 5 min using a microcentrifuge (Sigma 1–14, Germany). From the supernatant, 100 µL was carefully transferred into a 0.2 mL sterile microtube as extracted DNA. The assessment of DNA purity was performed by determining the A260/A280 and A230/A280 ratios using a Nanodrop 2000 UV–Vis spectrophotometer (Thermo Scientific, USA).

### Confirmation of E. coli isolates

All presumptive *E. coli* isolates were confirmed through the amplification of the *16 S rRNA* gene using polymerase chain reaction (PCR). The oligonucleotide primer sequences used were designed previously (Table 1) [23]. PCR amplification was performed using the Veriti™ 96-Well Thermal Cycler (Applied Biosystems, USA) with an initial denaturation step at 95 °C for 5 min, followed by 30 cycles of denaturation at 95 °C for 30 s, annealing at 58 °C for 30 s, and extension at 72 °C for 30 s. A final extension step was conducted at 72 °C for 5 min. The DNA template from the *E. coli* ATCC 25,922 strain served as the positive control, while distilled water was used as the negative control.

**Table 1:** The sequence of oligonucleotide primers

Primers’ name	Sequence (5’−3’)	Annealing (℃)	Product size (bp)	Reference
*E. coli* identification				
16 S rRNA-F 16 S rRNA-R	CCCCCTGGACGAAGACTGAC ACCGCTGGCAACAAAGGATA	58	401	[9]
Resistance genes				
blaCTX-M-F blaCTX-M-R	CGCTATTGCGATGTGCAG ACCTGCGATATCGTTGGT	63	550	[19]
blaTEM-F blaTEM-R	GAGTATTCAACATTTCCGTGTC TAATCAGTGAGGCACCTATCTC	43	848	
blaSHV-F blaSHV-R	AAGATCCACTATCGCCAGCAG ATTCAGTTCCGTTTCCCAGCGG	60	231	
blaOXA-48-F blaOXA-48-R	GCGTGGTTAAGGATGAACAC CATCAAGTTCAACCCAACCG	52	438	[20]
blaIMP-F blaIMP-R	GGAATAGAGTGGCTTAAYTCTC GGTTTAAYAAAACAACCACC		232	
blaVIM-F blaVIM-R	GATGGTGTTTGGTCGCATA CGAATGCGCAGCACCAG		390	
blaKPC-F blaKPC-R	CGTCTAGTTCTGCTGTCTTG CTTGTCATCCTTGTTAGGCG		798	
blaNDM-F blaNDM-R	GGTTTGGCGATCTGGTTTTC CGGAATGGCTCATCACGATC		621	
blaSPM-F blaSPM-R	AAAATCTGGGTACGCAAACG ACATTATCCGCTGGAACAGG		271	
mcr1-F mcr1-R	AGTCCGTTTGTTCTTGTGGC AGATCCTTGGTCTCGGCTTG	58	320	[21]
mcr2-F mcr2-R	CAAGTGTGTTGGTCGCAGTT TCTAGCCCGACAAGCATACC		715	
Sequence types				
ST69-F ST69-R	ATCTGGAGGCAACAAGCATA AGAGAAAGGGCGTTCAGAAT	60	104	[22]
ST73-F ST73-R	TGGTTTTACCATTTTGTCGGA GGAAATCGTTGATGTTGGCT		490	
ST95-F ST95-R	ACTAATCAGGATGGCGAGAC ATCACGCCCATTAATCCAGT		200	
ST131-F ST131-R	GACTGCATTTCGTCGCCATA CCGGCGGCATCATAATGAAA		310	
Phylogroups				
ArpA-F ArpA-R	AACGCTATTCGCCAGCTTGC TCTCCCCATACCGTACGCTA	59	400	[14]
chuA-F chuA-R	ATGGTACCGGACGAACCAAC TGCCGCCAGTACCAAAGACA		288	
yjaA-F yjaA-R	CAAACGTGAAGTGTCAGGAG AATGCGTTCCTCAACCTGTG		211	
TspE4.C2-F TspE4.C2-R	CACTATTCGTAAGGTCATCC AGTTTATCGCTGCGGGTCGC		152	

### Antimicrobial susceptibility testing

The Kirby-Bauer disc diffusion method was employed to assess the susceptibility of *E. coli *isolates to 20 antibiotics, as recommended by the Clinical and Laboratory Standards Institute (CLSI, 2024) guidelines [24]. The antibiotics tested included ampicillin (AMP, 10 µg), cefazolin (CZO, 30 µg), ceftazidime (CAZ, 30 µg), cefotaxime (CTX, 30 µg), ceftriaxone (CRO, 30 µg), cefepime (CPM, 30 µg), aztreonam (AZT, 30 µg), imipenem (IMP, 10 µg), meropenem (MER, 10 µg), amoxicillin/clavulanate (AMX, 20/10 µg), ampicillin/sulbactam (SAM, 10/10 µg), ticarcillin/clavulanate (TIM, 75/10 µg), fosfomycin (FOX, 200 µg), ciprofloxacin (CIP, 5 µg), tetracycline (TET, 30 µg), gentamicin (GEN, 10 µg), amikacin (AMK, 30 µg), chloramphenicol (CHL, 30 µg), and trimethoprim/sulfamethoxazole (SXT, 1.25/23.75 µg) (Mast Group, UK). The *E. coli *ATCC 25,922 strain was used as quality control. Additionally, susceptibility to colistin was determined by assessing the minimum inhibitory concentration (MIC) using the microbroth dilution method. For this purpose, a MIC range of colistin (Sigma-Aldrich Co., St. Louis, MO, USA) was prepared from 0.125 to 128 µg/mL in 96-well U-shaped microplates containing cation-adjusted Mueller-Hinton broth (CAMHB) (Merk, Germany). The eleventh and twelfth columns served as positive control (containing only CAMHB and the tested bacteria) and negative control (filled with sterile distilled water instead of bacteria), respectively. MDR isolates were defined as those resistant to at least one antibiotic in three or more antibiotic families. Furthermore, XDR isolates were identified as those non-susceptible to at least one antibiotic in all but two or fewer antibiotic families [25].

### ESBL detection

The potential for ESBL production was evaluated in isolates resistant to at least one of the tested third-generation cephalosporins and/or aztreonam antibiotics using the comparison disc diffusion method as suggested by CLSI [24]. To this end, a suspension equivalent to the 0.5 McFarland standard (1–1.5 × 10^8^ CFU/mL) was prepared from an overnight culture of the tested isolate. This suspension was then cultured to Mueller-Hinton agar (MHA) (Merk, Germany) using a sterile swab. Ceftazidime (CAZ, 30 µg) and cefotaxime (CTX, 30 µg) discs were placed adjacent to ceftazidime/clavulanate (CAZ/CV, 30/10 µg) and cefotaxime/clavulanate (CTX/CV, 30/10 µg) discs (Mast Group, UK) on the MHA culture, spaced 22–24 mm apart. Incubation was conducted at 35 ± 2 °C for 18–20 h. Positive results were indicated by an inhibition zone of ≥ 5 mm around each combined disc compared to the corresponding disc alone. *Klebsiella pneumoniae* ATCC 700,603 was used as the positive control.

### Evaluation carbapenemase production

Carbapenem-resistant *E. coli* isolates were selected to evaluate potential carbapenemase enzyme production using the modified Carbapenem Inactivation Method (mCIM). Additionally, the EDTA- modified Carbapenem Inactivation Method (eCIM) method was implemented to identify isolates producing Metallo-beta-lactamases (MBLs). A 1 µL loopful of fresh bacterial culture was inoculated into a tube containing 2 mL of TSB medium. A meropenem disk was then added to the tube, and incubation occurred at 35 ± 2 °C for 4 h ± 15 min. For the eCIM method, 20 µL of 0.5 M EDTA was added to the tube. After this period, the disks were transferred to the center of MHA plates that had been freshly inoculated with the carbapenem-susceptible *E. coli* ATCC 25,922 strain at a 0.5 McFarland suspension. The plates were subsequently incubated at 35 ± 2 °C for 16 to 20 h. Results were interpreted as follows: negative, ≥ 19 mm; positive, 6 to 15 mm; or intermediate (considered positive) if pinpoint colonies were observed within a 16 to 18 mm zone. For the eCIM method, a zone of inhibition around the eCIM disk of ≥ 5 mm in diameter compared to that of the mCIM disk indicated a positive result. Additionally, *Klebsiella pneumoniae* ATCC BAA-1705 and ATCC BAA-1706 strains were used as positive and negative controls, respectively.

### Molecular detection of resistance genes

PCR amplification was performed to assess the presence of three ESBL genes (*bla*TEM, *bla*CTX-M, and *bla*SHV), five carbapenemase genes (*bla*OXA-48, *bla*KPC, *bla*VIM, *bla*IMP, and *bla*NDM), and two plasmid-mediated colistin-resistance genes (*mcr*1 and *mcr*2) among all *E. coli* isolates, as well as carbapenem-resistant and colistin-resistant *E. coli* isolates, respectively. The PCR reaction was prepared in a final volume of 25 µL, which included 12.5 µL of 2× PCR Master Mix (Amplicon, Denmark), 1 µL of each primer (10 µM), 2 µL of template DNA (100 ng/µL), and 8.5 µL of nuclease-free water. An archived positive bacterial strain and distilled water were used as positive and negative controls, respectively. The oligonucleotide sequences of the primers used are listed in Table 1.

### Phylogroups analysis

A quadruplex PCR assay was established to detect four genes (*arpA*, *chuA*, *yjaA*, and *TspE4.C2*) in *E. coli* isolates. The oligonucleotide primer sequences, and PCR conditions were adopted from Clermont et al. (as shown in Table 1). Phylogenetic classification of the *E. coli *isolates was performed using the Clermont et al. (2013) quadruplex PCR method, which discriminates among phylogroups A, B1, B2, C, D, E, F, and Clade I [14].

### Prevalence of sequence types

Four main STs, including ST69, ST73, ST95, and ST13, were identified in *E. coli *isolates using multiplex PCR, as previously described by Doumith et al. [22]. The oligonucleotide sequences of the primers are illustrated in Table 1.

### Statistical analysis

Statistical analysis for this study was performed using SPSS Statistics version 22.0 (SPSS Inc., Chicago, Illinois, USA). The evaluation employed both Chi-square tests and Fisher’s exact tests, considering a *p*-value of less than 0.05 as statistically significant.

## Results

In total, 180 colonies, consisting of 90 colonies from 18 influent HWW samples and 90 colonies from effluent HWW samples, were collected for the identification of possible *E. coli* isolates. Sixty-eight *E. coli* isolates were identified based on standard biochemical tests and confirmed through amplification of the *16 S rRNA* gene. Of the 68 *E. coli* isolates, 35 (51.5%) were obtained from effluent HWW samples, while 33 (48.5%) were isolated from influent HWW samples. The distribution of *E. coli* isolates from various sources is presented in Table 2.

**Table 2:** The prevalence of resistant *E. coli* isolates according to different sampling sources (*N* = 68)

Antibiotics	Hospitals’ Name					
	Nemazee (*N* = 23)		Faghihi (*N* = 21)		Hafez (*N* = 24)	
	IF (*N* = 12)	EF (*N* = 11)	IF (*N* = 11)	EF (*N* = 10)	IF (*N* = 10)	EF (*N* = 14)
AMP (*N* = 66)	12 (18.2%)	11 (16.7%)	11 (16.7%)	9 (13.6%)	9 (13.6%)	14 (21.2%)
AMC/CV (*N* = 59)	12 (20.3%)	8 (13.6%)	11 (18.6%)	7 (11.9%)	9 (15.3%)	12 (20.3%)
CAZ (*N* = 57)	12 (21.1%)	8 (14%)	11 (19.3%)	8 (14%)	6 (10.5%)	12 (21.1%)
CTX (*N* = 58)	11 (19%)	9 (15.5%)	11 (19%)	8 (13.8%)	6 (10.3%)	13 (22.4%)
CZO (*N* = 59)	11 (18.6%)	10 (16.9%)	11 (18.6%)	8 (13.7%)	7 (11.9%)	12 (20.3%)
CRO (*N* = 57)	11 (19.3%)	9 (15.8%)	11 (19.3%)	8 (14%)	6 (10.5%)	12 (21.1%)
CPM (*N* = 49)	8 (16.3%)	7 (14.3%)	11 (22.4%)	7 (14.3%)	6 (12.2%)	10 (20.4%)
FOX (*N* = 15)	2 (13.3%)	1 (6.7%)	5 (33.3%)	2 (13.3%)	3 (20%)	2 (13.3%)
AZT (*N* = 50)	9 (18%)	8 (16%)	10 (20%)	7 (14%)	6 (12%)	10 (20%)
IMP (*N* = 4)	1 (25%)	0	1 (25%)	1 (25%)	0	1 (25%)
MRO (*N* = 9)	1 (11.1%)	0	3 (33.3%)	3 (33.3%)	0	2 (22.2%)
CIP (*N* = 36)	7 (19.4%)	5 (13.9%)	10 (27.8%)	4 (11.1%)	3 (8.3%)	7 (19.4%)
TET (*N* = 44)	8 (18.2%)	6 (13.6%)	9 (20.5%)	6 (13.6%)	8 (18.2%)	7 (15.9%)
SXT (*N* = 52)	10 (19.1%)	8 (15.4%)	11 (21.2%)	7 (13.5%)	8 (15.4%)	8 (15.4%)
CLF (*N* = 13)	1 (7.6%)	0	5 (38.5%)	2 (15.4%)	2 (15.4%)	3 (23.1%)
GEN (*N* = 17)	3 (13%)	3 (13%)	7 (30.4%)	4 (17.4%)	1 (4.4%)	5 (21.7%)
AMK (*N* = 21)	2 (9.5%)	3 (14.3%)	9 (42.9%)	3 (14.3%)	2 (9.5%)	2 (9.5%)
SAM (*N* = 58)	11 (19%)	8 (13.8%)	10 (17.2%)	9 (15.5%)	8 (13.8%)	12 (20.7%)
TIM (*N* = 53)	10 (18.9%)	8 (15.1%)	7 (13.2%)	8 (15.1%)	8 (15.1%)	12 (22.6%)
COL (*N* = 22)	1 (4.5%)	2 (9.1%)	8 (36.4%)	4 (18.2%)	3 (13.6%)	4 (18.2%)
Resistance phenotype						
ESBL (*N* = 39)	8 (20.5%)	5 (12.8%)	10 (25.6%)	5 (12.8%)	6 (15.4%)	5 (12.8%)
MDR (*N* = 55)	9 (16.4%)	8 (14.5%)	11 (20%)	9 (16.4%)	8 (14.5%)	10 (18.2%)
XDR (*N* = 15)	1 (6.7%)	0	9 (60%)	2 (13.3%)	1 (6.7%)	2 (13.3%)

*Abbreviation:* *IF* Influent, *EF* Effluent, *AMP* Ampicillin, *AMC/CV* Amoxicillin/clavulanate, *CZO* Cefazolin, *CAZ* Ceftazidime, *CTX* Cefotaxime, *CRO* Ceftriaxone, *CPM* Cefepime, *FOX* Fosfomycin, *AZT* Aztreonam, *IMP* Imipenem, *MER* Meropenem, *CIP* Ciprofloxacin, *TET* Tetracycline, *SXT* Trimethoprim/sulfamethoxazole, *CHL* Chloramphenicol, *GEN* Gentamicin, *AMK* Amikacin, *SAM* Ampicillin/sulbactam, *TIM* Ticarcillin/clavulanate, *COL* Colistin, *ESBL *Extended-spectrum β-lactamase, *MDR* Multi-drug resistant, *XDR* Extensively-drug resistant

###  Antimicrobial susceptibility testing

**Fig. 1:**
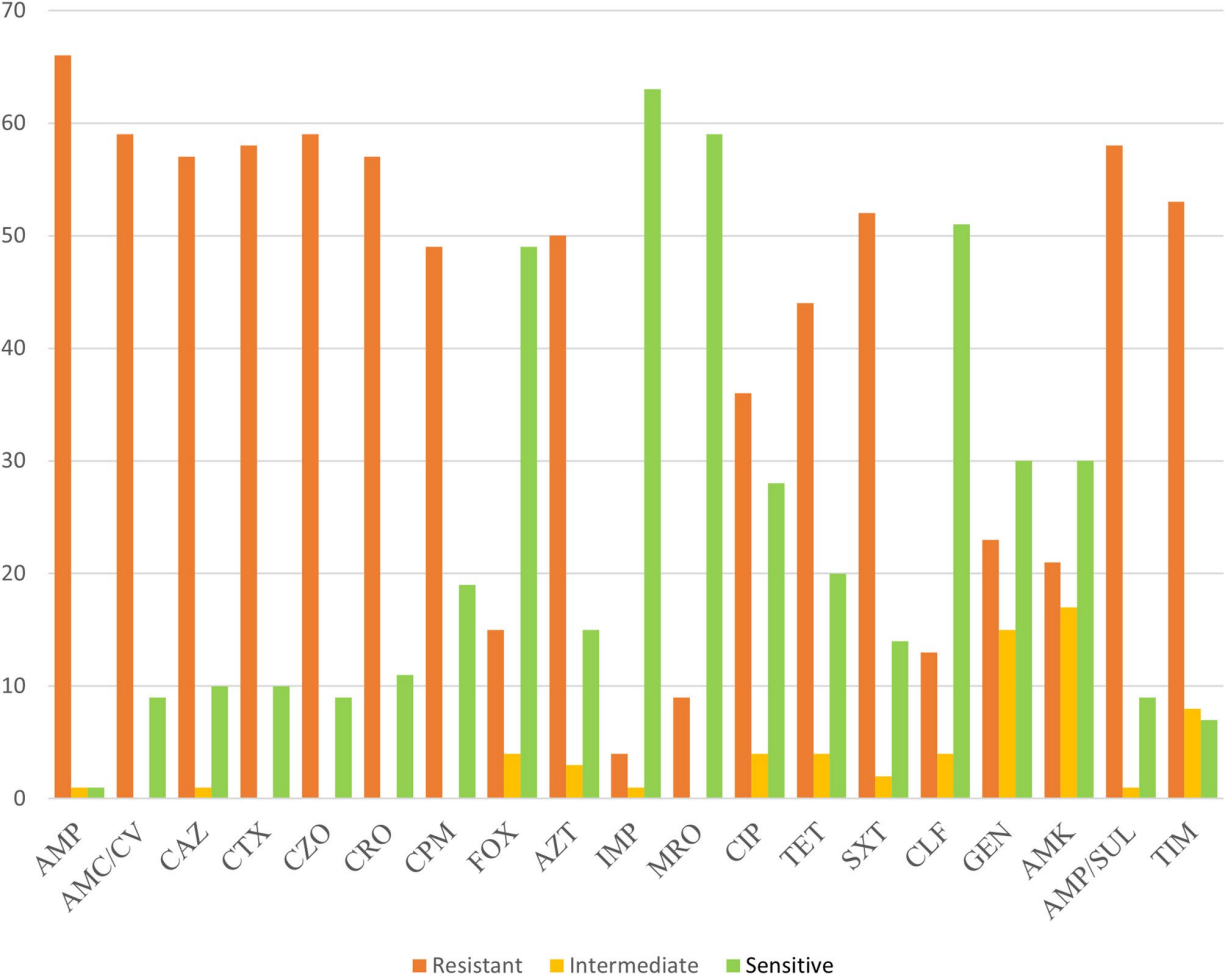
Antimicrobial susceptibility testing pattern among *E. coli* isolates from hospital wastewater (*N* = 68)

**Fig. 2:**
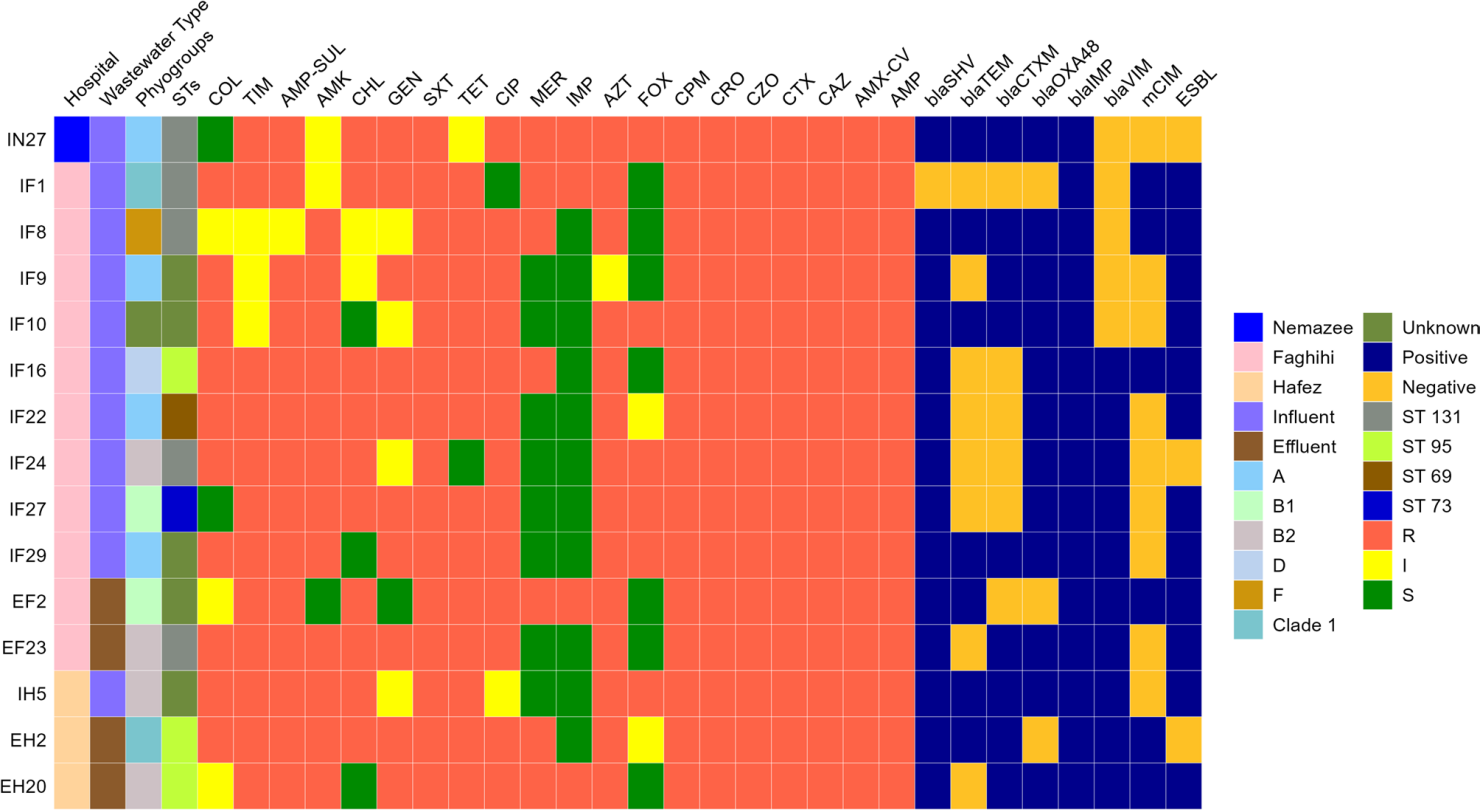
Characteristics of XDR *E. coli* isolates from influent and effluent hospital wastewater samples

The highest resistance was observed against ampicillin (66/68 isolates, 97.1%), cefazolin and amoxicillin/CV (59/68 isolates, 86.8%), and cefotaxime and ampicillin/sulbactam (58/68 isolates, 85.3%). In contrast, the lowest resistance rates were recorded against imipenem, meropenem, and chloramphenicol, with frequencies of 4/68 isolates (5.9%), 9/68 isolates (13.2%), and 13/68 isolates (19.1%), respectively (Fig. 1). Among the 68 *E. coli* isolates examined, 32/68 (47%) were resistant to colistin, with the majority of these isolates (19/68, 27.9%) exhibiting a MIC of 1 µg/mL. Additionally, the MIC50 and MIC90 were determined to be 1 µg/mL and 16 µg/mL, respectively. The prevalence of MDR and XDR *E. coli* isolates was 55/68 (80.9%) and 15/68 (22.1%), respectively. XDR *E. coli* isolates were characterized as shown in Fig. 2. The prevalence of resistant isolates according to different sampling sources is detailed in Tables 2 and 3.

**Table 3:** Correlation of resistant *E. coli* isolates according to different sampling sources (*N* = 68)

Antibiotics	Hospitals			*p* value^*^	Wastewater types		*p* value
	Nemazee 23 (%)	Faghihi 21 (%)	Hafez 24 (%)		Influent 33 (%)	Effluent 35 (%)	
AMP (66)	23 (100%)	20 (95.2%)	23 (95.8%)	0.525	32 (97%)	34 (97.1%)	0.739
AMC/CV (59)	20 (87%)	18 (85.7%)	21 (87.5%)	1	32 (97%)	27 (77.1%)	0.017
CZA (57)	20 (87%)	19 (90.5%)	18 (75%)	0.613	29 (87.9%)	28 (80%)	0.307
CTX (58)	20 (87%)	19 (90.5%)	19 (79.2%)	0.635	28 (84.8%)	30 (85.7%)	0.92
CZO (59)	21 (90.3%)	19 (90.5%)	19 (79.2%)	0.487	29 (87.9%)	30 (85.7%)	0.539
CRO (57)	20 (87%)	19 (90.5%)	18 (75%)	0.389	28 (84.8%)	29 (82.9%)	0.824
CPM (49)	15 (65.2%)	18 (85.7%)	16 (66.7%)	0.243	25 (75.8%)	24 (68.6%)	0.509
FOX (15)	3 (13%)	7 (33.3%)	5 (20.8%)	0.539	10 (30.3%)	5 (14.3%)	0.21
AZT (50)	17 (73.9%)	17 (81%)	16 (66.7%)	0.836	25 (78.8%)	25 (71.4%)	0.646
IMP (4)	1 (4.3%)	2 (9.5%)	1 (4.2%)	0.678	2 (6.1%)	2 (5.7%)	1
MRO (9)	1 (4.3%)	6 (28.6%)	2 (8.3%)	0.066	4 (12.1%)	5 (14.3)	0.539
CIP (36)	12 (52.2%)	14 (66.7%)	10 (41.7%)	0.335	20 (60.6%)	16 (45.7%)	0.433
TET (44)	14 (60.9%)	15 (71.4%)	15 (62.5%)	0.445	25 (78.8%)	19 (54.3%)	0.138
SXT (52)	18 (78.3%)	18 (85.7%)	16 (66.7%)	0.445	29 (87.9%)	23 (65.7%)	0.074
CLF (13)	1 (4.3%)	7 (33.3%)	5 (20.8%)	0.027	8 (24.2%)	5 (14.3%)	0.57
GEN (23)	6 (26.1%)	11 (52.4%)	6 (25%)	0.257	11 (33.3%)	12 (34.3%)	0.52
AMK (21)	5 (21.7%)	12 (57.1%)	4 (16.7%)	0.022	13 (39.4%)	8 (22.9%)	0.105
AMP/SUL (58)	19 (82.6%)	19 (90.5%)	20 (83.3%)	0.426	29 (87.9%)	29 (82.9%)	0.478
TIM (53)	18 (78.3%)	15 (71.4%)	20 (83.3%)	0.829	25 (78.8%)	28 (80%)	0.969
COL	3 (13%)	12 (57.1%)	7 (29.2%)	0.016	12 (36.4%)	10 (28.6%)	0.766
Resistance phenotype							
ESBL	13 (56.5%)	15 (71.4%)	11 (45.8%)	0.222	24 (72.7%)	15 (42.9%)	0.013
MDR	17 (73.9%)	20 (95.2%)	18 (75%)	0.130	28 (84.8%)	(27 (77.1%)	0.419
XDR	1 (4.3%)	11 (52.4%)	3 (12.5%)	< 0.001	11 (33.3%)	4 (11.4%)	0.029

*Abbreviation:* *IF* Influent, *EF* Effluent, *AMP* Ampicillin, *AMC/CV* Amoxicillin/clavulanate, *CZO* Cefazolin, *CAZ* Ceftazidime, *CTX* Cefotaxime, *CRO* Ceftriaxone, *CPM* Cefepime, *FOX* Fosfomycin, *AZT* Aztreonam, *IMP* Imipenem, *MER* Meropenem, *CIP* Ciprofloxacin, *TET* Tetracycline, *SXT* Trimethoprim/sulfamethoxazole, *CHL* Chloramphenicol, *GEN* Gentamicin, *AMK* Amikacin, *SAM* Ampicillin/sulbactam, *TIM* Ticarcillin/clavulanate, *COL* Colistin, *ESBL* Eextended-spectrum β-lactamase, *MDR* Multi-drug resistant, *XDR* Extensively-drug resistant.

^*^p value ≤ 0.05 is significant

### ESBL and carbapenemase production

Among the 68 *E. coli* isolates investigated for ESBL production, 39 isolates (57.4%) were identified as ESBL producers. Additionally, of the 9 *E. coli* isolates assessed for carbapenemase production (which were resistant to meropenem and/or imipenem), 8 isolates (88.9%) tested positive in the mCIM. However, none were positive in the eCIM test, suggesting that the carbapenemase enzymes present in these isolates are likely serine-based (e.g., Class A or Class D serine carbapenemases), rather than MBLs. Furthermore, 31/39 (79.5%) ESBL-EC isolates were classified as MDR, while 12/39 (30.8%) were XDR. Additionally, all 8 (100%) carbapenemase-producing *E. coli* isolates were MDR, 5/8 (62.5%) were XDR, and 7/8 (87.5%) were ESBL producers.

### Detection of resistance genes

The PCR amplification products were visualized using gel electrophoresis (Supplementary File 1). The *bla*TEM gene was the most predominant ESBL-mediated gene, observed in 27/68 isolates (39.7%), followed by *bla*CTX-M at a rate of 23/68 (33.8%) and *bla*SHV at 2/68 (2.9%). Among the 8 carbapenemase-producing *E. coli* isolates, 3 (37.5%) tested positive for the *bla*OXA-48 gene. Additionally, one isolate was positive for the *bla*IMP gene, and another was positive for the *bla*VIM gene. The *bla*NDM, *bla*KPC, *bla*SPM, *mcr*1, and *mcr*2 genes were not detected. The most frequent pattern of co-harboring resistance genes was observed in 12/68 *E. coli* isolates that co-harbored *bla*TEM and *bla*CTX-M (Fig. 2). Notably, among these isolates, 11/12 (91.7%) were classified as MDR and 5/12 (41.7%) as XDR.

### Phylogroup analysis

**Fig. 3:**
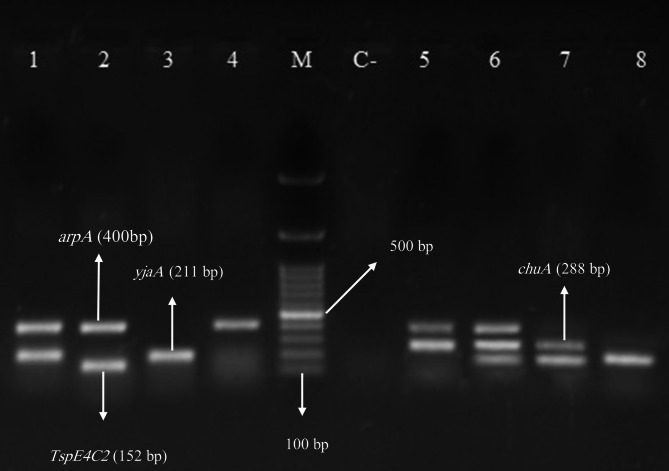
The multiplex PCR amplification products for determining phylogroups were displayed using agarose gel electrophoresis (1.5% agarose). Lane M: DNA marker ladder (100 bp plus). Lane 1: phylogroup A or C (arpA + iv, chuA -ve, yjaA + ve, TspE4C2 -ve). Lane 2: phylogroup B1 (arpA + iv, chuA -ve, yjaA -ve, TspE4C2 + ve). Lane 3: Clade I (arpA -iv, chuA -ve, yjaA + ve, TspE4C2 -ve). Lane 4: phylogroup A (arpA + iv, chuA -ve, yjaA -ve, TspE4C2 -ve). Lane C-: control negative. Lane 5: D or E (arpA + iv, chuA + ve, yjaA -ve, TspE4C2 -ve). Lane 6: phylogroup E (arpA + iv, chuA + ve, yjaA + ve, TspE4C2 -ve). Lane 7: phylogroup B2 (arpA -iv, chuA + ve, yjaA + ve, TspE4C2 -ve). Lane 8: phylogroup F (arpA + iv, chuA + ve, yjaA -ve, TspE4C2 -ve)

According to phylogrouping (Fig. 3), the majority of isolates (15/68, 22.1%) belonged to phylogroup B2. Additionally, 14/68 isolates (20.6%) were categorized as phylogroup A, and 10/68 isolates (14.7%) were classified as Clade I.

### Prevalence of sequence types

The prevalence of four STs of *E. coli*, namely ST69, ST73, ST95, and ST131, were investigated among all isolates. The most prevalent ST was ST131, occurring in 23/68 isolates (33.8%), followed by ST73 with 13/68 isolates (19.1%), and both ST69 and ST95, each with 7/68 isolates (10.3%). Additionally, 18/68 isolates (26.5%) tested negative for any of the four investigated STs.

### Statistical analysis

According to the statistical results, the prevalence of resistance to chloramphenicol, amikacin, colistin, and XDR *E. coli* isolates from Nemazee, Faghihi, and Hafez HWW were as follows: 4.3% vs. 33.3% vs. 20.8% (*p* ≤ 0.001), 21.7% vs. 57.1% vs. 16.7% (*p* = 0.18), 13% vs. 57.1% vs. 29.2% (*p* = 0.16), and 4.3% vs. 52.4% vs. 12.5% (*p* ≤ 0.001). These results indicate a significant difference in the prevalence of resistance among *E. coli* isolates from Faghihi Hospital.

Additionally, the prevalence of resistance to amoxicillin/CV, ESBL-EC, and XDR *E. coli* isolates among influent and effluent HWW samples was 97% vs. 77.1% (*p* = 0.016), 33.3% vs. 11.4% (*p* = 0.029), and 72.7% vs. 42.9% (*p* = 0.013), respectively. These results demonstrate a significantly higher frequency of resistance profiles in isolates from influent compared to effluent samples.

There was a positive correlation between ESBL-EC isolates and resistance to amikacin when compared to susceptible isolates (46.2% vs. 10.3%; *p* = 0.005). Although a significant relationship was not observed between ESBL-EC and MDR and non-MDR isolates (79.5% vs. 82.8%; *p* = 0.734), significant differences were found between ESBL-EC and XDR isolates (30.8% vs. 10.3%; *p* = 0.045) when compared to non-XDR isolates.

The presence of the *bla*TEM gene among gentamicin-resistant *E. coli* isolates was significantly higher than in susceptible isolates (55.6% vs. 19.5%, *p* = 0.007). Additionally, the prevalence of the *bla*CTX-M gene varied among resistant *E. coli* isolates to cefepime (87% vs. 64.4%, *p* = 0.05), aztreonam (91.3% vs. 64.4%, *p* = 0.04), and *E. coli* (73.9% vs. 48.9%, *p* = 0.048) when compared with susceptible and non-ESBL-producing isolates. Table 4 represents the prevalence of MDR, XDR, and ESBL-EC isolates categorized by various phylogroups and STs.

**Table 4:** The prevalence of MDR, XDR, and ESBL producing *E. coli* isolates according to the different phylogroups and sequence types

Phylogroups	MDR		Total	*p* value	XDR		Total	*p* value	ESBL		Total	*p* value
	+ve	-ve			+ve	-ve			+ve	-ve		
A	12	2	14	0.623	4	10	14	0.486	7	7	14	0.274
B1	5	2	7		2	5	7		4	3	7	
B2	12	3	15		4	11	15		11	4	15	
A/C	5	1	6		0	6	6		1	5	6	
E	3	0	3		1	2	3		2	1	3	
D/E	4	4	8		0	8	8		6	2	8	
F	4	1	5		1	4	5		2	3	5	
Clade I	10	0	10		3	7	10		6	4	10	
Sequence typs												
ST69	4	3	7	0.23	1	6	7	0.318	6	1	7	0.179
ST73	11	2	13		1	12	13		9	4	13	
ST95	7	0	7		3	4	7		4	3	7	
ST131	20	3	23		5	18	23		9	14	23	
Unknown	13	5	18		5	13	18		11	7	18	

*Abbreviation:* *MDR* Multi-drug resistant, *XDR* Extensively-drug resistant, *ESBL* Extended-spectrum β-lactamase, *+ve* Positive, *-ve* Negative

^*^p value ≤ 0.05 is significant

No additional significant correlations were found between the variables.

## Discussion

AMR is now recognized as one of the most significant global health concerns, transitioning us from the ‘golden era of antibiotics’ to an ‘era with no antibiotics’ [26]. Combating the development of AMR requires enormous collaborative efforts under a ‘One Health’ approach, which encompasses an entire interaction among human medicine, veterinary science, agriculture, and environmental health. The development of AMR occurs through the dissemination of ARGs and environmental pollution among bacteria [27].

Wastewater, particularly HWW, has been identified as a significant pathway for the dissemination of pathogens and ARGs. Although recent publications have reported a high frequency of resistant bacteria and ARG transmission among humans, livestock, and the environment [28–30], there is a lack of robust documentation regarding the circulation of resistant bacteria and ARGs among humans and veterinary sources in environments such as HWW [31]. Therefore, in the current study, the prevalence of AMR patterns was assessed both phenotypically and genotypically among *E. coli* isolates from HWW.

Generally speaking, among the tested β-lactam antibiotic group, we found a high resistance rate to ampicillin (66/68, 97.1%), as well as to extended- and non-extended-spectrum cephalosporins, including cefazolin (59/68, 86.8%), cefotaxime (58/68, 85.3%), ceftazidime and ceftriaxone (57/68, 83.8%), and cefepime (49/68, 72.1%). In contrast, carbapenems, including imipenem and meropenem, demonstrated the lowest resistance rates, with frequencies of 4/68 (5.9%) and 9/68 (13.2%) for *E. coli *isolates, respectively. Our findings of resistance to cephalosporins were generally higher than those reported in previous studies [32, 33]; however, their findings on resistance rates against carbapenems were not significantly different from the results of the current study.

Acquisition of resistance to β-lactam antibiotics occurs due to the production of β-lactamase enzymes, with ESBLs being the most prevalent. *Klebsiella pneumoniae* (*K. pneumoniae*) and *E. coli *are recognized as the most predominant producers of ESBLs. This assertion is supported by several systematic reviews and meta-analyses, which indicate a pooled prevalence of ESBL-EC of 21.1% (95% CI: 19.1% − 23.2%) and 17.6% (95% CI: 15.3% − 19.8%) among inpatients and healthy individuals, respectively [34]; additionally, the overall pooled prevalence was reported as 33% (95% CI: 28.2% − 38.1%) for *E. coli* and 32.7% (95% CI: 28.6% − 37.1%) for *K. pneumoniae *[35]. In Iran, a study conducted at an urban wastewater treatment plant in Ardabil reported that 7/34 (20.6%) of *E. coli *isolates were ESBL producers, with 6/34 (17.6%) being ESBL-only and 1/34 (2.9%) producing both ESBL and AmpC enzymes [36]. This prevalence is significantly lower than the 57.4% observed in our study, indicating a higher burden of ESBL-EC in our sampled wastewater. Also, it is higher than the earlier survey by La Thi Quynh et al. in Vietnam, which reported a frequency of 115/265 (43.3%) ESBL-EC isolates [33]. However, more recent studies have documented a higher incidence of ESBL-EC isolates, with rates of 53/73 (72.6%) in Czechia [37]and 102/109 (93.5%) in Burkina Faso [38], compared to our findings. Globally, a meta-analysis encompassing 57 studies reported a pooled prevalence of ESBL-producing *Enterobacteriaceae* in wastewater at 24.81%, with *E. coli *being the most prevalent species [39]. This global average further underscores the elevated prevalence found in our study. However, as AmpC enzymes are not inhibited by clavulanic acid, their presence may have gone undetected, potentially leading to an underestimation of β-lactamase-mediated resistance in isolates co-producing ESBL and AmpC enzymes. Nonetheless, detection of AmpC β-lactamases was beyond the scope of the current study, which was specifically designed to identify ESBL-EC based on CLSI-recommended phenotypic and molecular methods [24]. Future studies should incorporate specific phenotypic or molecular assays (e.g., cefoxitin screening, AmpC multiplex PCR) to provide a more comprehensive resistance profile. Despite this limitation, our findings indicate that the prevalence of ESBL-EC in wastewater samples is notably higher than national and global averages reported in similar contexts. This elevated prevalence may reflect regional differences in antibiotic usage, wastewater management practices, and antimicrobial stewardship.

Interestingly, a positive correlation has been observed between ESBL-EC isolates and MDR; the majority of ESBL-EC isolates were found to be resistant to various classes of antibiotics, particularly fluoroquinolones, tetracyclines, aminoglycosides, and trimethoprim/sulfamethoxazole [40]. Although our results demonstrated a significant relationship only between ESBL-EC and amikacin-resistant isolates (*p* = 0.005), it is noteworthy that 55 out of 68 (80.9%) *E. coli* isolates were categorized as MDR, and 15 out of 68 (22.1%) were classified as XDR. In addition, the observed higher prevalence of XDR isolates at Faghihi Hospital compared to Nemazee Hospital may be influenced by various hospital-specific factors such as size, bed occupancy, patient population, and antibiotic stewardship programs. Unfortunately, detailed data on these parameters were not available in this study, limiting our ability to explore their impact.

The prevalence of MDR *E. coli* isolates in the present study is consistent with an earlier investigation by Redha et al. in Kuwait, which reported MDR *E. coli *isolates from HWW at a rate of 91.4% among 128 tested isolates; however, their finding of XDR isolates (1.4%) contradicts our results [41]. These discrepancies may be attributed to differing geographical patterns of AMR, changes in AMR patterns over time, the use of various methods for determining AMR, and other influencing factors.

In general, comparison of the resistance frequencies to amoxicillin/CV, ESBL-EC, and XDR *E. coli* isolates revealed significantly higher resistance rates among isolates from influent HWW samples compared to effluent samples: 97% vs. 77.1% (*p* = 0.016), 33.3% vs. 11.4% (*p* = 0.029), and 72.7% vs. 42.9% (*p*= 0.013), respectively. This finding is supported by previous study that reported higher resistance rates among isolates from influent HWW compared to effluent samples [33]. Conversely, Bozorgomid et al. reported a higher frequency of resistance among *E. coli *isolates from effluent HWW [42]. These discrepancies may be attributed to differences in wastewater treatment processes, seasonal variation during sampling, or variations in the methodologies used to assess resistance profiles.

The tracking of ESBL-mediated genes revealed that *bla*TEM was the most predominant gene, with a frequency of 27/68 (39.7%), while the prevalence of *bla*CTX-M was 23/68 (33.8%). These findings support earlier studies that reported a higher frequency of *bla*TEM compared to *bla*CTX-M, with rates of 97% vs. 64% [33], and 92% vs. 60% [42], respectively. However, other investigations have found *bla*CTX-M to be more common than *bla*TEM, with frequencies of 100% versus 79% [43], and 49% versus 2% [37], respectively. Although *bla*TEM and *bla*SHV genes were detected in our investigation, we acknowledge that not all variants of these genes encode ESBLs. For instance, *bla*TEM-1 and *bla*SHV-1 are formerly well-known narrow-spectrum β-lactamases and do not confer ESBL activity [44]. In the present study, subtyping or sequencing of these genes was not performed; therefore, we could not distinguish ESBL-associated variants from non-ESBL ones. This is a limitation in interpreting the molecular findings. However, it is important to note that all isolates harboring *bla*TEM or *bla*SHV genes were first confirmed as ESBL producers using the combination disk method, indicating functional ESBL activity. Furthermore, our methodological approach aligns with multiple peer-reviewed studies [45, 46] that similarly interpret *bla*TEM and *bla*SHV detection in phenotypically confirmed isolates as markers of ESBL production, especially in resource-limited settings.

Interestingly, although *bla*IMP and *bla*VIM genes were detected in one isolate each, these isolates tested negative by the phenotypic eCIM assay designed to detect MBL activity. This discrepancy could be due to several factors, including partial gene sequences, mutations affecting gene functionality, or lack of gene expression under the tested conditions. Such silent or non-expressed carbapenemase genes may not confer phenotypic resistance detectable by eCIM, highlighting the limitations of relying solely on phenotypic assays for carbapenemase detection. Therefore, combining molecular and phenotypic methods is crucial for accurately assessing the presence and expression of carbapenemase genes in environmental isolates [47].

Although phenotypic colistin resistance was confirmed in 32/68 isolates, the absence of *mcr*−1 and *mcr*−2 genes suggests that other mechanisms, such as chromosomal mutations in genes like *pmr*A, *pmr*B, or *mgr*B, may contribute to resistance [48]. These alternative mechanisms were not investigated in the present study, representing a limitation. Future research employing whole-genome sequencing or targeted molecular analyses is needed to better understand the full spectrum of colistin resistance in these isolates.

In the current study, the majority of isolates were assigned to ST131, accounting for 23/68 isolates (33.8%), followed by ST73, with 13/68 isolates (19.1%). This finding aligns with previous research by Davidova-Gerzova et al., which reported ST131 as the most predominant ST in HWW samples, with a frequency of 80/408 isolates (19.6%). However, in their report, the second most frequent STs was ST38 [37]. Typically, ST69, ST73, ST95, and ST131 are primarily associated with extraintestinal pathogenic *E. coli *(ExPEC) strains. Among these, ST131 is notably recognized for its MDR profile [22, 49]. Our results indicate that 20/55 (36.4%) of MDR isolates were assigned to ST131, as well as 5/15 (33.3%) of XDR isolates were classified as ST131.

The detection of ST131 in wastewater samples underscores its potential role in the dissemination of MDR pathogens within the community. ST131 is a globally recognized clone associated with urinary tract infections and bloodstream infections, often exhibiting resistance to multiple antibiotics [49]. Studies have demonstrated the presence of ST131 in various environmental sources, including wastewater treatment plants, indicating its widespread distribution beyond clinical settings [50, 51]. The repeated isolation of ST131 from wastewater suggests a continuous influx from human and possibly animal sources, reflecting its prevalence in the population. This environmental presence raises concerns about the potential for HGT of resistance determinants to other bacteria in aquatic ecosystems, posing risks to public health [52]. While our study did not directly link ST131 isolates from wastewater to clinical infections, the established association of this clone with human disease highlights the importance of monitoring its environmental reservoirs. Future research should focus on comparative genomic analyses between environmental and clinical ST131 isolates to elucidate transmission pathways and inform intervention strategies.

According to phylogroup analysis, the majority of *E. coli *isolates were categorized as belonging to phylogroup B2 (14/68, or 22.1%) and phylogroup A (14/68, or 20.6%), respectively. In contrast, Bozorgomid et al. reported that the most predominant phylogroups were B1 (28/60, 46.7%) and A (14/60, 23.3%), respectively [42]. Also, Kumar et al. [53] determined that more than 50% of 217 tested *E. coli* isolates from HWW and municipal wastewater (MWW) belonged to phylogroups B1 and A. Notably, the prevalence of A phylogroup isolates was significantly higher in HWW compared to MWW (*p* < 0.001). However, there are limited investigations available that report on the prevalence of different phylogroups among *E. coli* isolated from HWW.

This study faced several limitations that may impact the interpretation of the findings. Firstly, the research was conducted in only three major hospitals in Shiraz, which may limit the generalizability of the results to other healthcare settings. Secondly, the absence of municipal wastewater sampling and the analysis of genetic relatedness between isolates from hospital and municipal sources restricts the understanding of the broader environmental context. Additionally, the study focused on selected resistance genes, potentially overlooking other significant genetic determinants of antimicrobial resistance. The six-week sampling period did not account for seasonal variations, highlighting the need for future longitudinal studies to provide a more comprehensive view. Due to sample size constraints, only bivariate analyses were performed, limiting the ability to adjust for confounders and making causal inferences challenging. Therefore, the findings should be viewed as exploratory and hypothesis-generating, warranting further research in this area.

## Conclusion

This study identifies a notable prevalence of MDR, XDR, and ESBL-EC in HWW from Shiraz, Iran. Our results suggest that septic treatment may reduce some levels of antibiotic resistance, but measurable levels of AMR persist in effluents, indicating a need for improvements in wastewater treatment processes. Including additional hospitals in future research could provide a more comprehensive understanding of AMR dissemination and the effectiveness of treatment strategies. The identification of high-risk clones such as ST131 and the presence of phylogroup B2 may indicate that HWW could serve as a reservoir for certain clinically relevant resistance patterns. Although AmpC producers were not evaluated in this study, the documented prevalence of ESBL is of concern. These findings highlight the importance of routine monitoring of HWW and encourage enhancements in wastewater treatment practices within a One Health framework. Considering the reported use of antibiotics in veterinary settings in Iran, future research should consider integrating data from livestock and agriculture to further explore the potential for zoonotic transmission and its implications for environmental AMR.

## Supplementary Information

Supplementary Material 1

## Data Availability

All data generated or analyzed during this study are included in this published article.

## Abbreviations

*E. coli*: *Escherichia coli*
WHO: World health organization
AMR: Antimicrobial resistance
MDR: Multi-drug resistant
HWW: Hospital wastewater
ARGs: Antimicrobial resistance genes
ECDC: European centre for disease prevention and control
ESBLs: Extended-spectrum β-lactamases
ESBL-EC: Extended-spectrum β-lactamase-producing *E. coli*
XDR: Extensively-drug resistant
EMB: Eosin methylene blue
TSI: Triple sugar iron
TSB: Tryptic soy broth
PCR: Polymerase-chain reaction
CLSI: Clinical and laboratory standards institute
MIC: Minimum inhibitory concentration
CAMHB: Cation-adjusted Mueller-Hinton broth
MHA: Mueller-Hinton agar
mCIM: Modified carbapenem inactivation method
eCIM: EDTA-modified carbapenem inactivation method
ExPEC: Extraintestinal pathogenic *E. coli*
MHH: Municipal wastewater
