# Supplementary material for: High prevalence of MDR and XDR Escherichia coli in hospital wastewater from Shiraz, Iran: ESBL and carbapenemase production
Source: BMC Microbiol. 2025 Oct 9;25:647. doi: 10.1186/s12866-025-04398-2 (PMC12512558; doi:10.1186/s12866-025-04398-2)
Supplement: Supplementary file 1 — Supplementary Material 1 [file 12866_2025_4398_MOESM1_ESM.docx]

**Supplementary Figures:** The PCR amplified products were displayed using agarose gel electrophoresis (1.5% agarose).


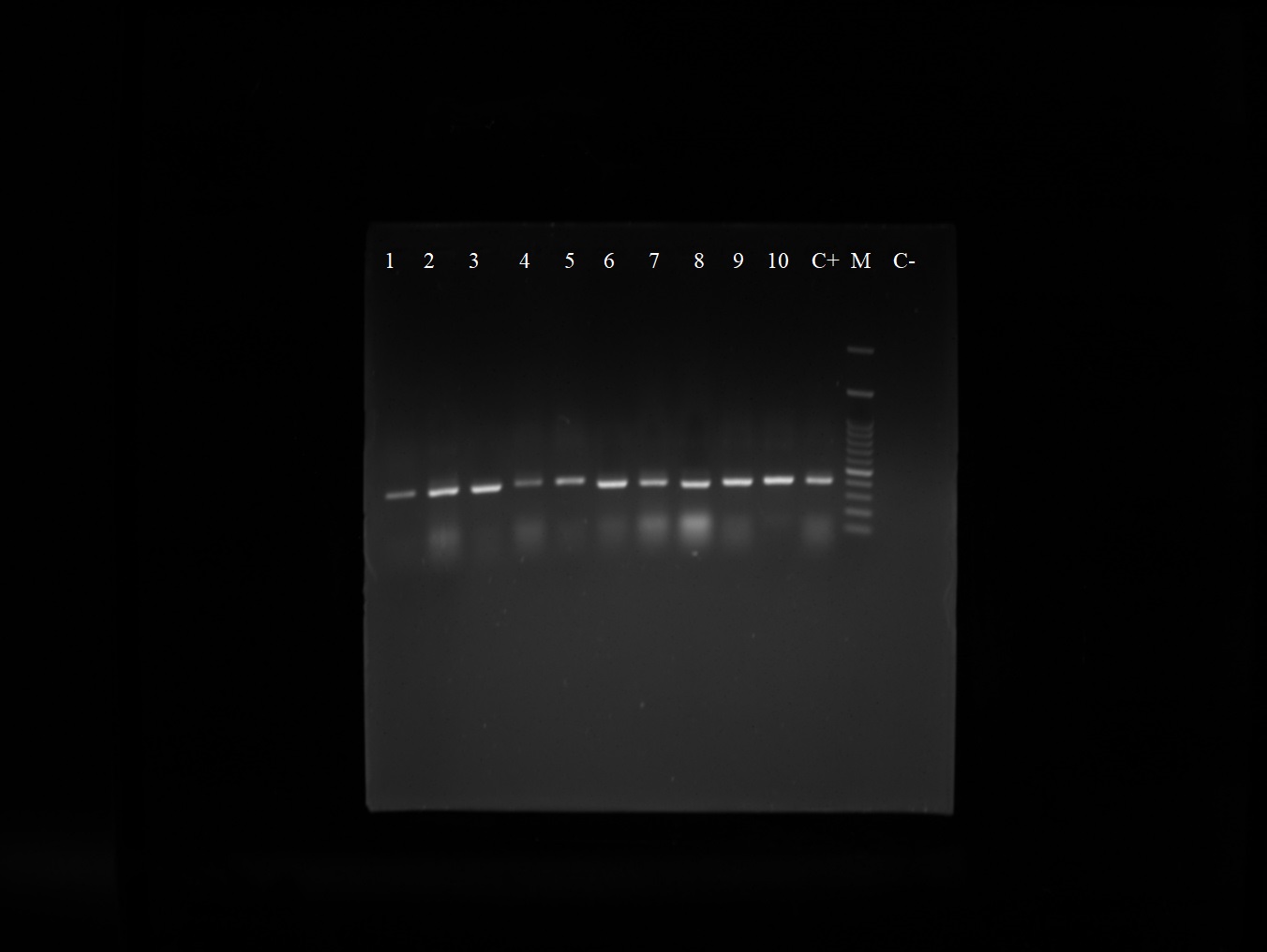


*16S rRNA* (401 bp)

500 bp

**Figure 1** Lane M: DNA marker (100 bp plus DNA Ladder), Lane: C+: control positive, Lane C-: negative control, Lanes 1–10: Detection of 16S rRNA gene for confirmation of E. coli isolates.


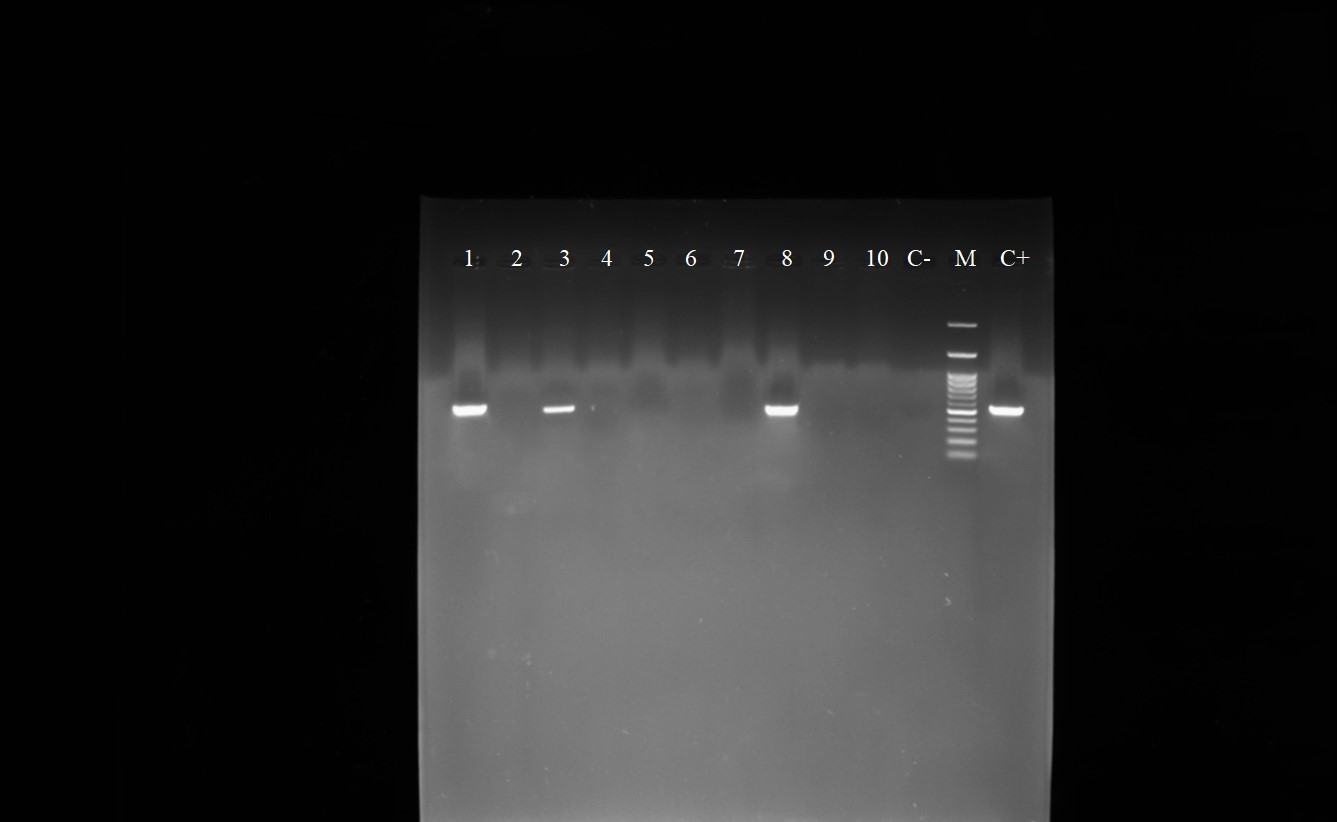


500 bp

*bla*CTX-M (550 bp)

**Figure 2** Lane M: DNA marker (100 bp plus DNA Ladder), Lane C-: negative control, Lane C+: positive control, Lanes 1,3, and 8: detection of blaCTX-M genes in E. coli isolates.


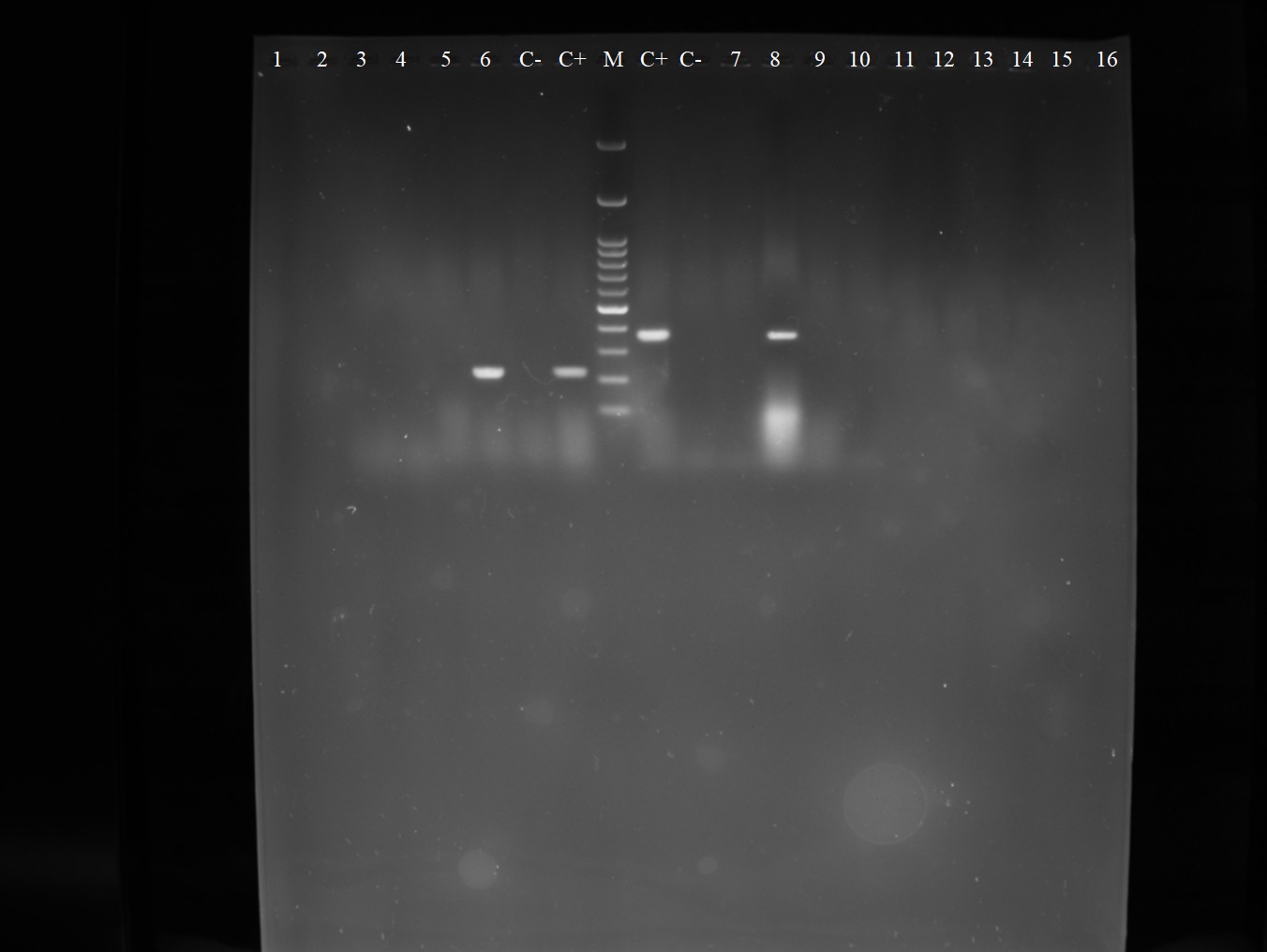


*bla*VIM (390 bp)

500 bp

*bla*IMP (232 bp)

**Figure 3** Lane M: DNA marker (100 bp plus DNA Ladder), Lane C-: negative control, Lane C+: positive control, Lane 6: Detection of blaIMP gene, Lane 8: Detection of blaVIM gene in carbapenem-resistant E. coli isolates.


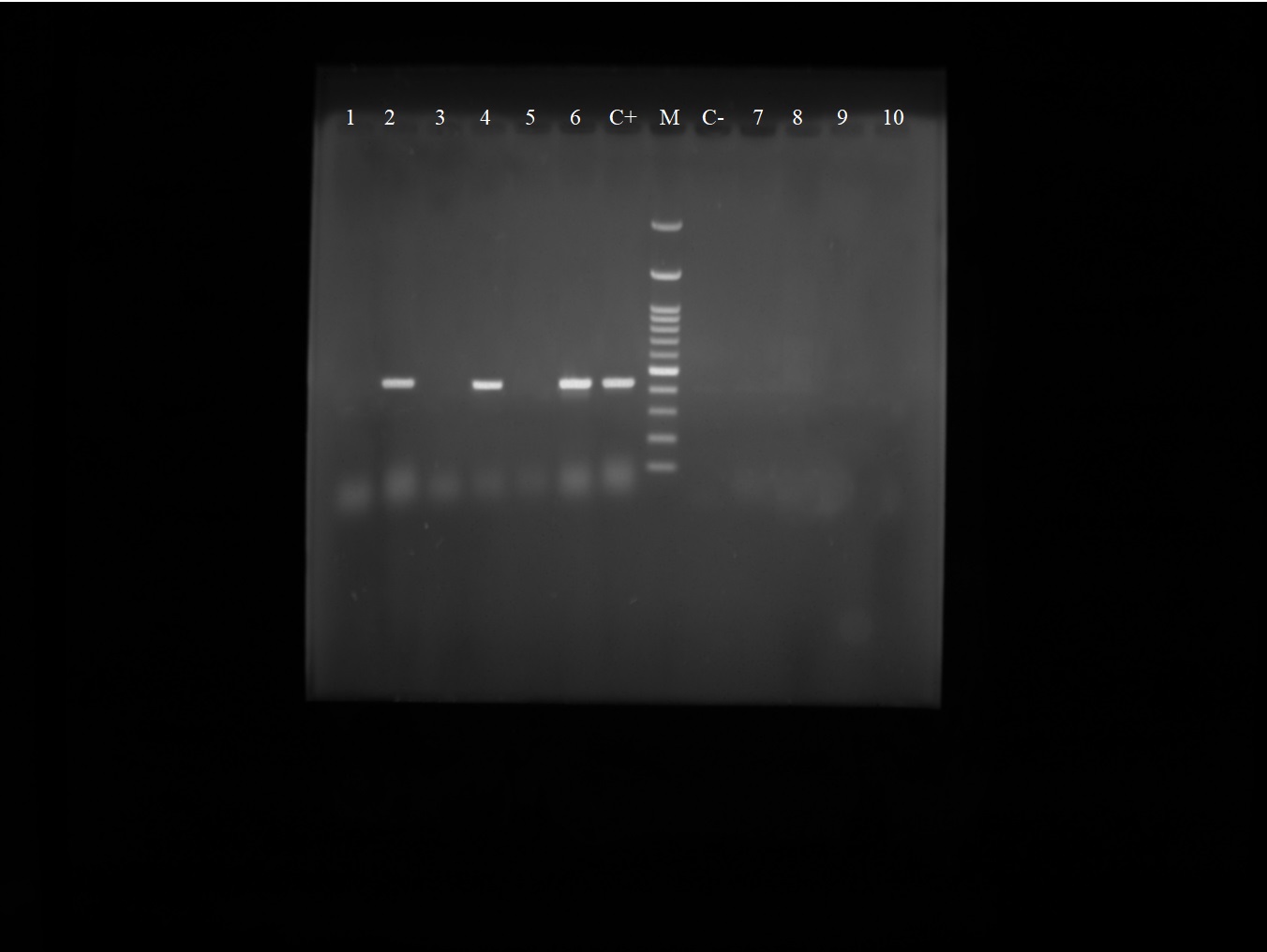


500 bp

*bla*OXA-48 (438 bp)

**Figure 4** Lane M: DNA marker (100 bp plus DNA Ladder), Lane C+: positive control, Lane C-: negative control, Lane 2, 4, and 6: Detection of blaOX-48 gene in carbapenem-resistant E. coli isolates.


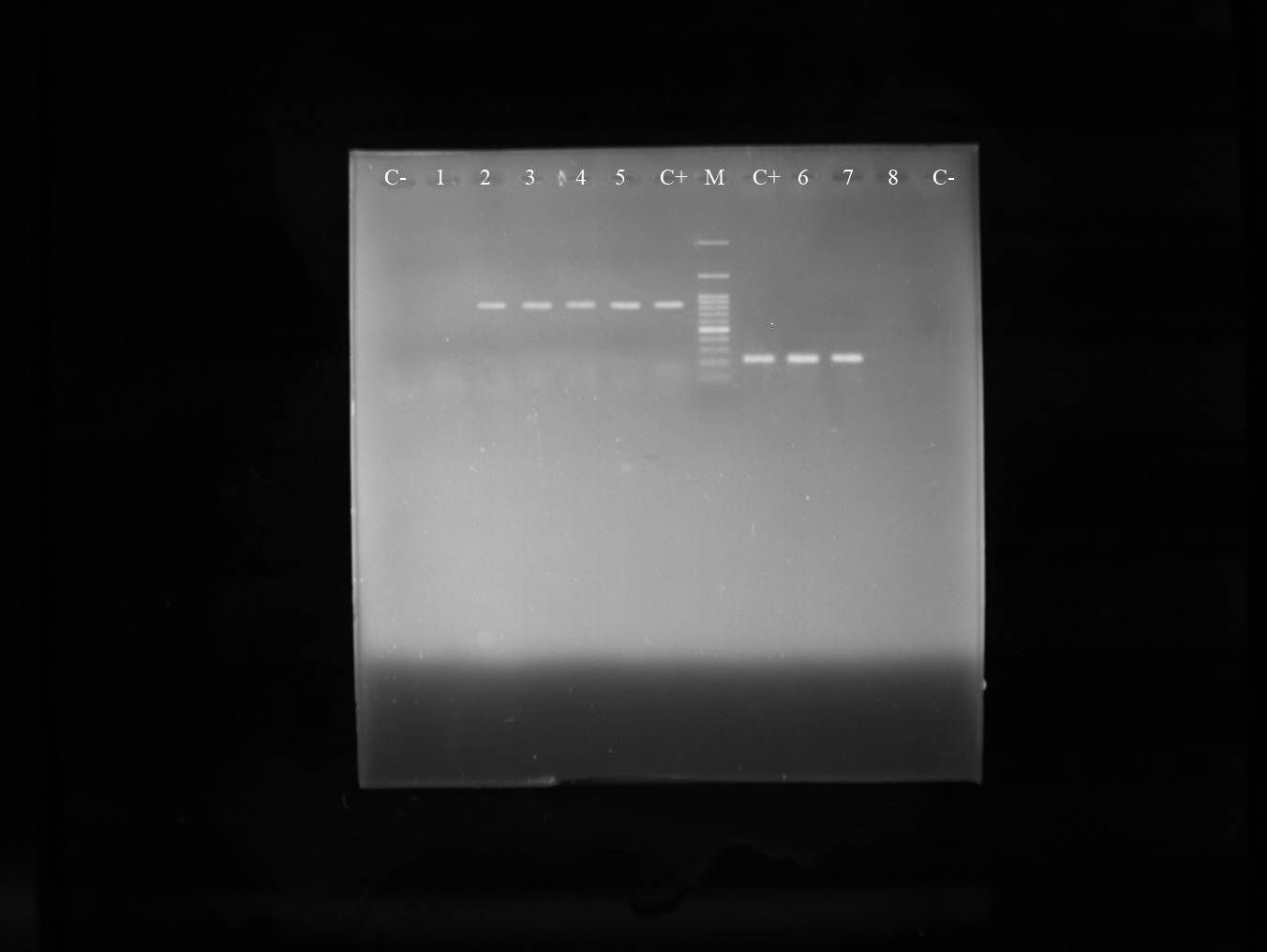


*bla*SHV (231 bp)

*bla*TEM (848 bp)

500 bp

**Figure 5** Lane M: DNA marker (100 bp plus DNA Ladder), Lane C-: negative control, Lane C+: positive control, Lanes 3-6: Detection of blaTEM gene, Lanes 7 and 8: detection of blaSHV in E. coli isolates.


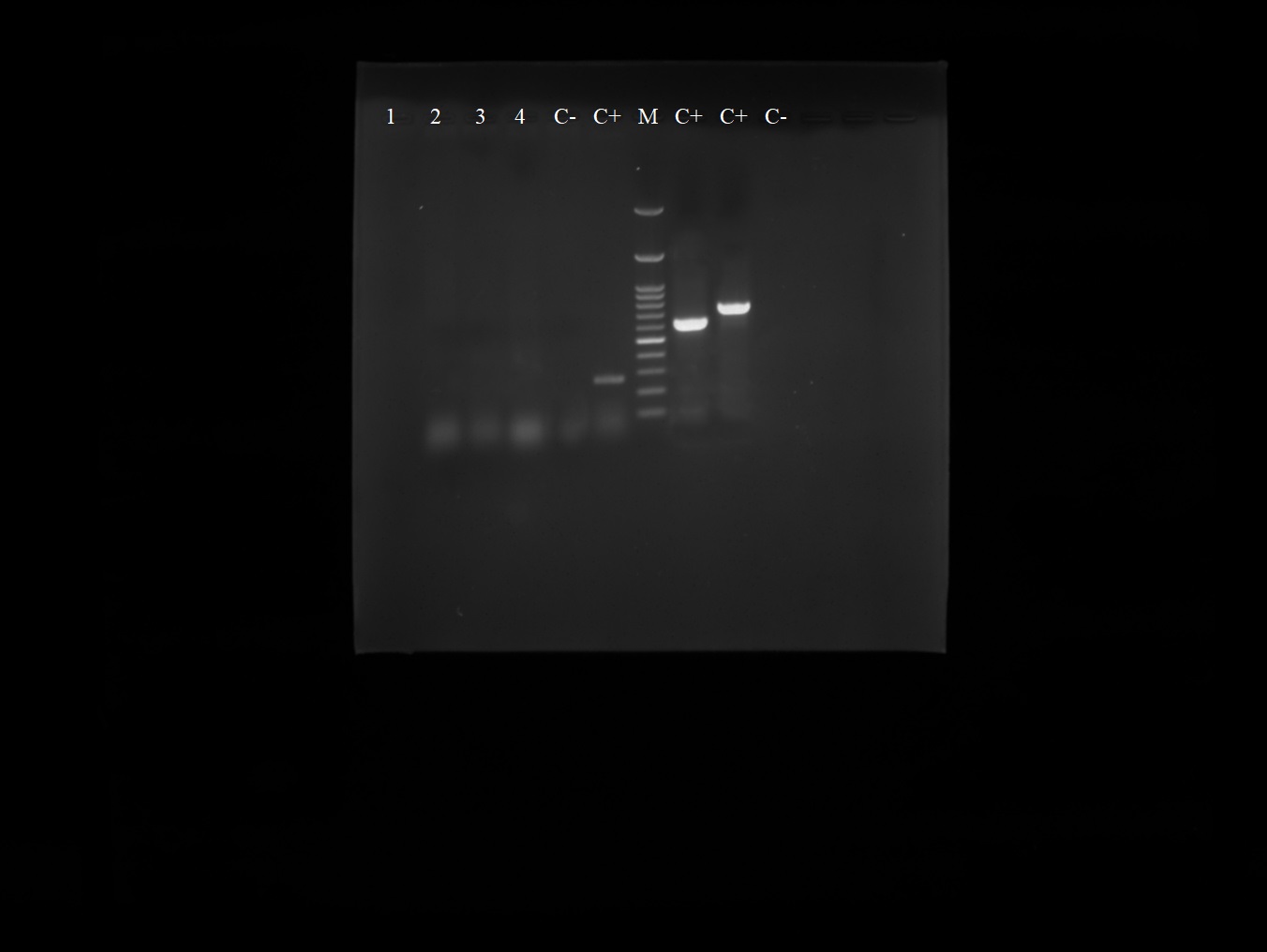


*bla*NDM (621 bp)

*bla*KPC (798 bp)

*bla*SPM (271 bp)

500 bp

**Figure 6** Lane M: DNA marker (100 bp plus DNA Ladder), Lane C-: negative control, Lane C+: positive control of the amplification of the blaSPM, blaNDM, and blaKPC genes.


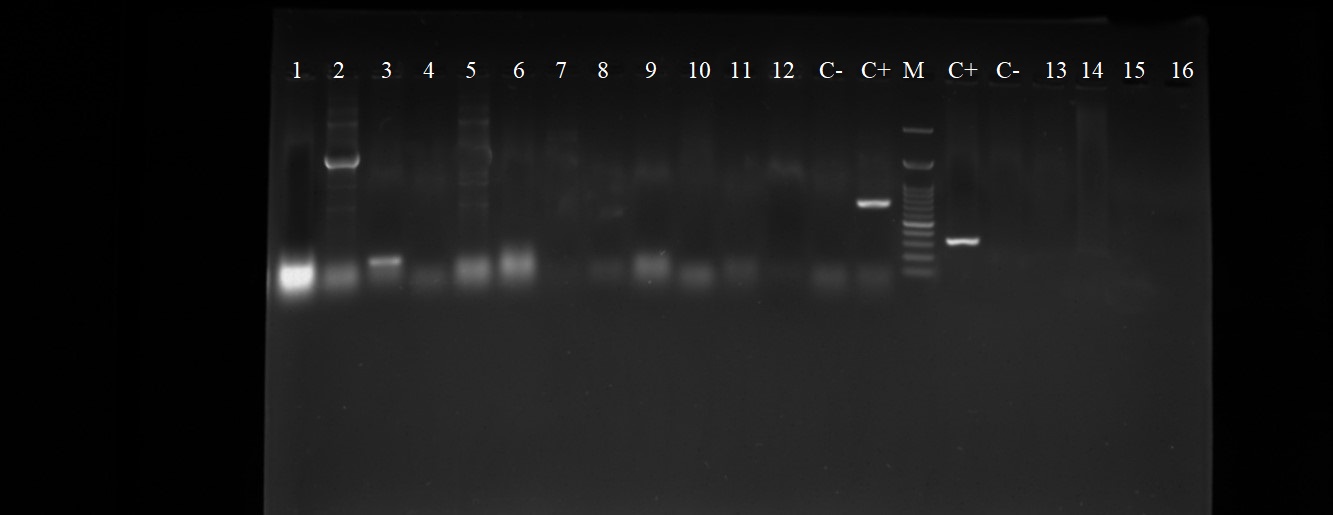


*mcr*1 (320 bp)

500 bp

*mcr*2 (715 bp)

**Figure 7** Lane M: DNA marker (100 bp plus DNA Ladder), Lane C-: negative control, Lane C+: positive control of the amplification of the mcr1 and mcr2 genes.


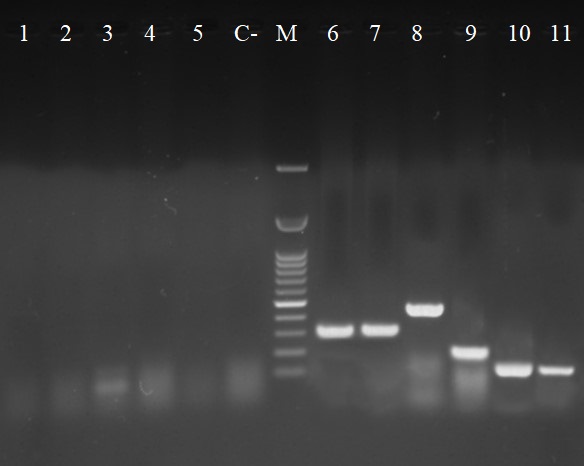


104 bp

200 bp

490 bp

310 bp

500 bp

**Figure 8** Lane M: DNA marker (100 bp plus DNA Ladder), Lane C-: negative control, Lanes 6 and 7: Detection of ST131, Lane 8: Detection of ST73, Lane 9: Detection of ST95, Lanes 10 and 11: Detection of ST69.
